# Supplementary material for: ATP hydrolysis-driven structural transitions within the S. cerevisiae Rad51 and Dmc1 nucleoprotein filaments
Source: bioRxiv. 2025 Mar 19:2025.03.19.644215. Preprint. [Version 1] doi: 10.1101/2025.03.19.644215 (PMC11957116; doi:10.1101/2025.03.19.644215)
Supplement: 1 [file NIHPP2025.03.19.644215V1-supplement-1.pdf]

bioRxiv preprint doi: https://doi.org/10.1101/2025.03.19.644215; this version posted March 19, 2025. The copyright holder for this preprint (which was not certified by peer review) is the author/funder, who has granted bioRxiv a license to display the preprint in perpetuity. It is made available under aCC-BY 4.0 International license.

Table S1. CryoEM parameters.

	Rad51_ADP PDB: 9NJK (EMD-49485)	Dmc1_ADP PDB: 9NJR (EMD-49488)
**Data collection and processing**		
Microscope	Titan Krios	Titan Krios
Voltage (keV)	300	300
Detector	K3	K3
Magnification	105,000	105,000
Voltage (kV)	300	300
Electron exposure ($e^-/Å^2$)	50	58
Defocus range (μm)	-0.75 to -1.75	-0.75 to -1.75
Pixel size (Å)	0.855	0.823
Initial particles picked	1,748,800	1,184,484
Final particles used	330,601	960,233
Map resolution (Å)<br>FSC threshold	3.37<br>0.143	2.7<br>0.143
Map resolution range (Å)	3.3-3.8	2.7-3.3
**Refinement**		
Model resolution (Å)<br>FSC threshold	3.4<br>0.143	2.8<br>0.143
Model composition<br>Non-hydrogen atoms<br>Protein residues<br>Ligands	 13,808<br>1,793<br>ADP, MG	 13,886<br>1,779<br>ADP, MG
R.m.s. deviations<br>Bond lengths (Å)<br>Bond angles (°)	 0.002<br>0.589	 0.004<br>0.628
Validation<br>MolProbity score<br>Clash score<br>Rotamer outliers (%)	 1.56<br>10.92<br>0	 1.78<br>13.67<br>0.07
Ramachandran plot<br>Favored (%)<br>Allowed (%)<br>Outliers (%)	 98.35<br>1.65<br>0	 97.25<br>2.75<br>0

bioRxiv preprint doi: https://doi.org/10.1101/2025.03.19.644215; this version posted March 19, 2025. The copyright holder for this preprint (which was not certified by peer review) is the author/funder, who has granted bioRxiv a license to display the preprint in perpetuity. It is made available under aCC-BY 4.0 International license.

Figure S1

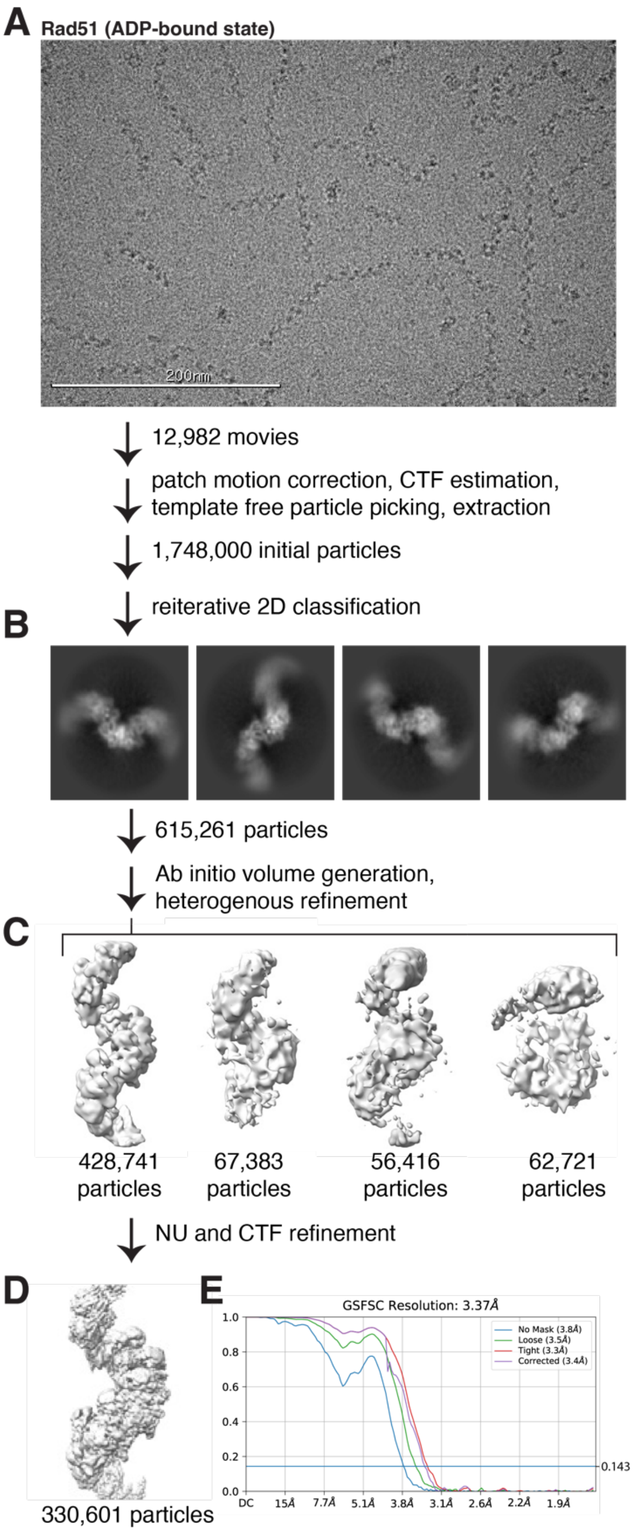

bioRxiv preprint doi: https://doi.org/10.1101/2025.03.19.644215; this version posted March 19, 2025. The copyright holder for this preprint (which was not certified by peer review) is the author/funder, who has granted bioRxiv a license to display the preprint in perpetuity. It is made available under aCC-BY 4.0 International license.

Figure S1. Cryo-EM image processing pipeline of the Rad51-ADP nucleoprotein filament (A) Representative micrograph used for the Rad51-ADP nucleoprotein filament data processing. **(B)** Representative 2D classes selected for 3D classification. **(C)** 3D map generation and refinements. **(D)** Final 3D map reconstruction for the Rad51-ADP nucleoprotein filament. **(E)** Fourier shell correlation curve of the final electron density map.

bioRxiv preprint doi: https://doi.org/10.1101/2025.03.19.644215; this version posted March 19, 2025. The copyright holder for this preprint (which was not certified by peer review) is the author/funder, who has granted bioRxiv a license to display the preprint in perpetuity. It is made available under aCC-BY 4.0 International license.

Figure S2

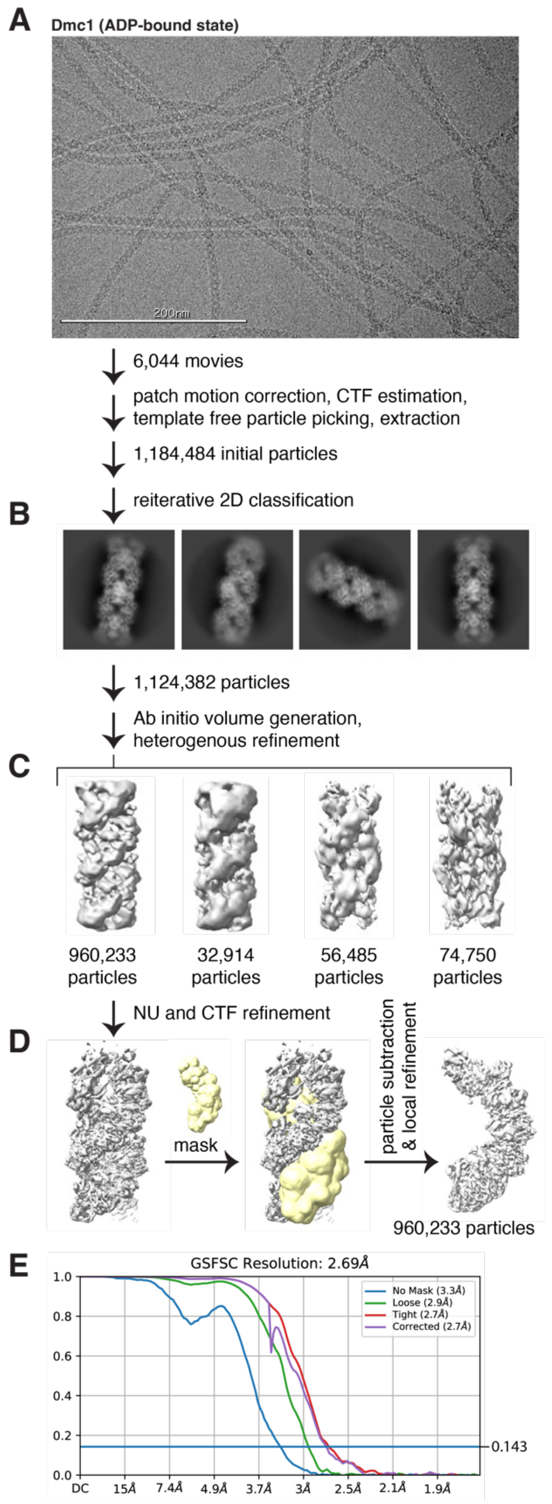

bioRxiv preprint doi: https://doi.org/10.1101/2025.03.19.644215; this version posted March 19, 2025. The copyright holder for this preprint (which was not certified by peer review) is the author/funder, who has granted bioRxiv a license to display the preprint in perpetuity. It is made available under aCC-BY 4.0 International license.

Figure S2. Cryo-EM image processing pipeline of the Dmc1-ADP nucleoprotein filament (A) Representative micrograph used for the Dmc1-ADP nucleoprotein filament data processing. **(B)** Representative 2D classes selected for 3D classification. **(C)** 3D map generation and refinements. **(D)** Final 3D map reconstruction for the Dmc1-ADP nucleoprotein filament. **(E)** Fourier shell correlation curve of the final electron density map.

bioRxiv preprint doi: https://doi.org/10.1101/2025.03.19.644215; this version posted March 19, 2025. The copyright holder for this preprint (which was not certified by peer review) is the author/funder, who has granted bioRxiv a license to display the preprint in perpetuity. It is made available under aCC-BY 4.0 International license.

Figure S3

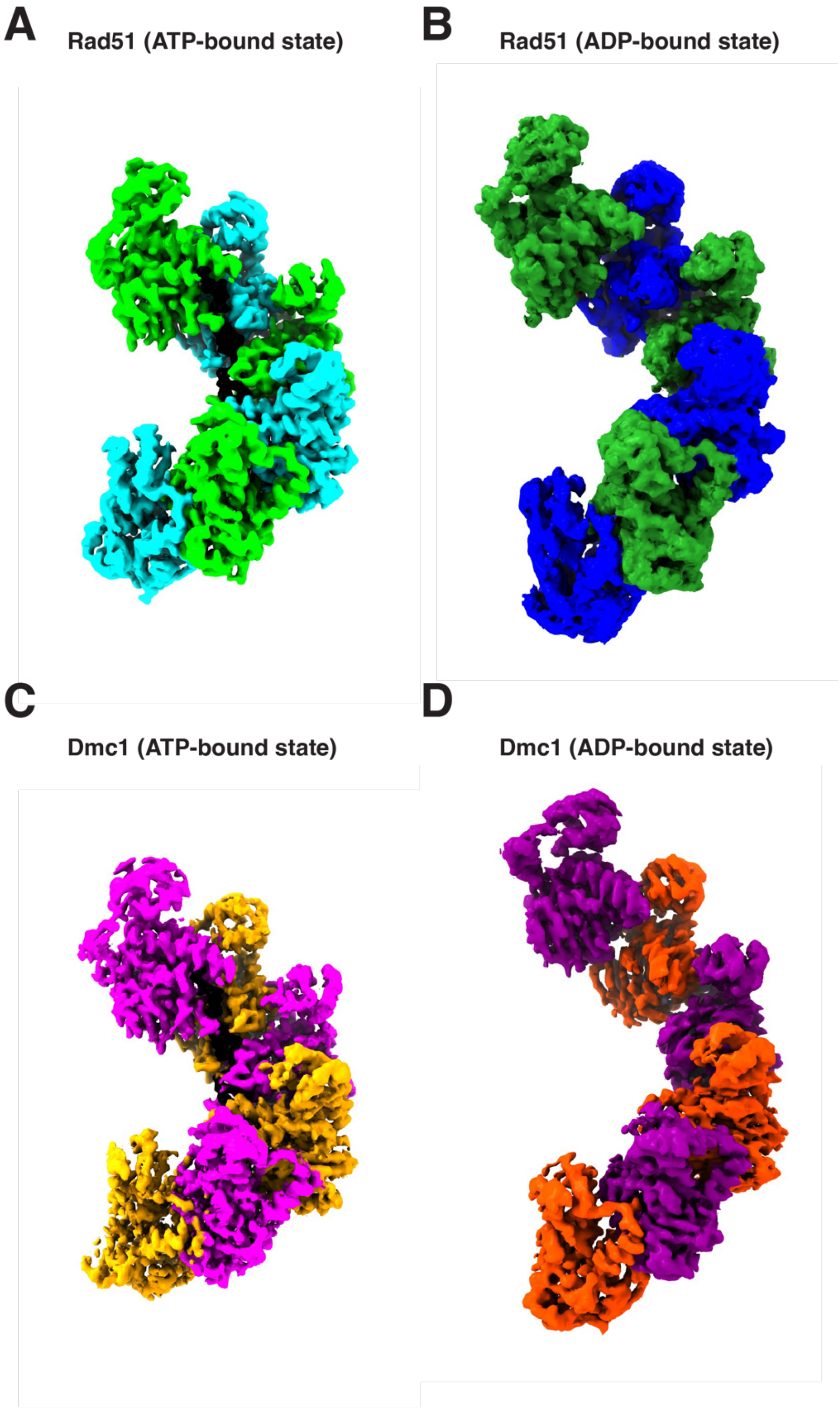

bioRxiv preprint doi: https://doi.org/10.1101/2025.03.19.644215; this version posted March 19, 2025. The copyright holder for this preprint (which was not certified by peer review) is the author/funder, who has granted bioRxiv a license to display the preprint in perpetuity. It is made available under aCC-BY 4.0 International license.

Figure S3. Cryo-EM density maps for the ATP- and ADP-bound nucleoprotein filaments. (A) Rad51 in the ATP bound state. (B) Rad51 in the ADP-bound state. (C) Dmc1 in the ATP bound state. (D) Dmc1 in the ADP-bound state. For each panel, a subsection of the nucleoprotein comprised of six Rad51 or Dmc1 monomers is shown, and the different protein monomers are highlighted in alternating colors. The ssDNA is shown in black.

bioRxiv preprint doi: https://doi.org/10.1101/2025.03.19.644215; this version posted March 19, 2025. The copyright holder for this preprint (which was not certified by peer review) is the author/funder, who has granted bioRxiv a license to display the preprint in perpetuity. It is made available under aCC-BY 4.0 International license.

Figure S4

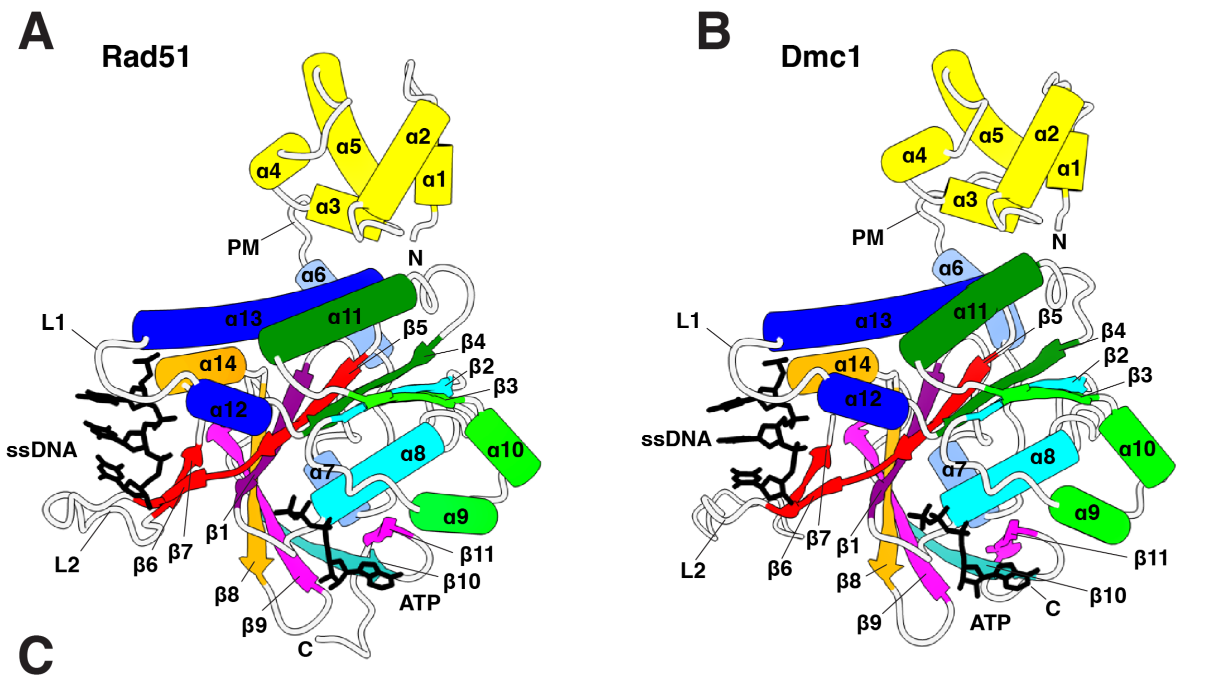

C

```
                                 α1        α2         α3     α4
ScRad51   MSQVQEQHISESQLQYGNGSLMSTVPADLSQSVVDGNGNGSSEDIEATNGSGDGGGLQEQ   60
ScDmc1    ------------------------------------------------------------   0

ScRad51   AEAQGEMEDEAYDEAALGSFVPIEKLQVNGITMADVKKLRESGLHTAEAVAYAPRKDLLE   120
ScDmc1    ----MSVTGTEIDSDTAKNILSVDELQNYGINASDLQKLKSGGIYTVNTVLSTTRRHLCK   56
              .: .    *. :   .:: :::**  **. :*::**:..*::*.::*   : *:.* :

            α5               PM   α6              α7
ScRad51   IKGISEAKADKLLNEAARLVPMGFVTAADFHMRRSELICLTTGSKNLDTLLGGGVETGSI   180
ScDmc1    IKGLSEVKVEKIKEAAGKIIQVGFIPATVQLDIRQRVYSLSTGSKQLDSILGGGIMTMSI   116
          ***:**.*..:*: : *.::: :**: *:      *..: .*:****:**::****: * **

          β1 Walker A  α8                  β2          α9
ScRad51   TELFGEFRTGKSQLCHTLAVTCQIPLDIGGGEGKCLYIDTEGTFRPVRLVSIAQRFGLDP   240
ScDmc1    TEVFGEFRCGKTQMSHTLCVTTQLPREMGGGEGKVAYIDTEGTFRPERIKQIAEGYELDP   176
          **:***** **:*:.***.** *:* ::******  ********** *: .**: : ***

          α10  β3      α11              β4 Walker B α12   L1   α13
ScRad51   DDALNNVAYARAYNADHQLRLLDAAAQMMSESRFSLIVVDSVMALYRTDFSGRGELSARQ   300
ScDmc1    ESCLANVSYARALNSEHQMELVEQLGEELSSGDYRLIVVDSIMANFRVDYCGRGELSERQ   236
          :..* **:**** *::**:.*::  .: :*.. : ******:** :*.*:.****** **

               α13             β5     β6     L2        β7   α14   β8
ScRad51   MHLAKFMRALQRLADQFGVAVVVTNQVVAQVDGGMAF-NPDPKKPIGGNIMAHSSTTRLG   359
ScDmc1    QKLNQHLFKLNRLAEEFNVAVFLTNQVQSDPGASALFASADGRKPIGGHVLAHASATRIL   296
           :* :.:  *:***::*.***.:**** ::  ...   *  .  * :*****:::**:*:**:

          β8    β9          β10    β11
ScRad51   FKKGKGCQRLCKVVDSPCLPEAECVFAIYEDGVGDPREEDE   400
ScDmc1    LRKGRGDERVAKLQDSPDMPEKECVYVIGEKGITDSSD---   334
          ::**:* :*:.*: *** :** ***:.* *.*: *   :
```

bioRxiv preprint doi: https://doi.org/10.1101/2025.03.19.644215; this version posted March 19, 2025. The copyright holder for this preprint (which was not certified by peer review) is the author/funder, who has granted bioRxiv a license to display the preprint in perpetuity. It is made available under aCC-BY 4.0 International license.

Figure S4. Diagrams of Rad51 and Dmc1 secondary structure topology. (A) Ribbon diagram of *S. cerevisiae* Rad51 in the ATP-bound state (PDB ID: 9D46)[44] showing the number designation for each alpha helix and beta strand. Also highlighted are the Walker A and B motifs, DNA binding loops L1 and L2, and the FxxA polymerization motif (PM). The bound ssDNA and ATP are shown in black. **(B)** Ribbon diagram of *S. cerevisiae* Dmc1 in the ATP-bound state (PDB ID: 9D4N)[44] showing the number designation for each alpha helix and beta strand. **(C)** Sequence alignment of *S. cerevisiae* Rad51 (UniProt ID: P25454) and Dmc1(UniProt ID: P25453) (residues in red are small, hydrophobic, or both [A, V, I, L, M, F, P]; residues in blue have acidic side chains [D, E]; residues in magenta have basic side chains [K, R]; green residues correspond to all others [H, S, T, N, Q, C, G, Y, W]; "*"indicates identical residues in Rad51 and Dmc1; "**.**" indicates residues have weakly similar properties; "**:**" indicates strongly similar residues). The sequence was aligned using Clustal Omega [62].

bioRxiv preprint doi: https://doi.org/10.1101/2025.03.19.644215; this version posted March 19, 2025. The copyright holder for this preprint (which was not certified by peer review) is the author/funder, who has granted bioRxiv a license to display the preprint in perpetuity. It is made available under aCC-BY 4.0 International license.

Figure S5

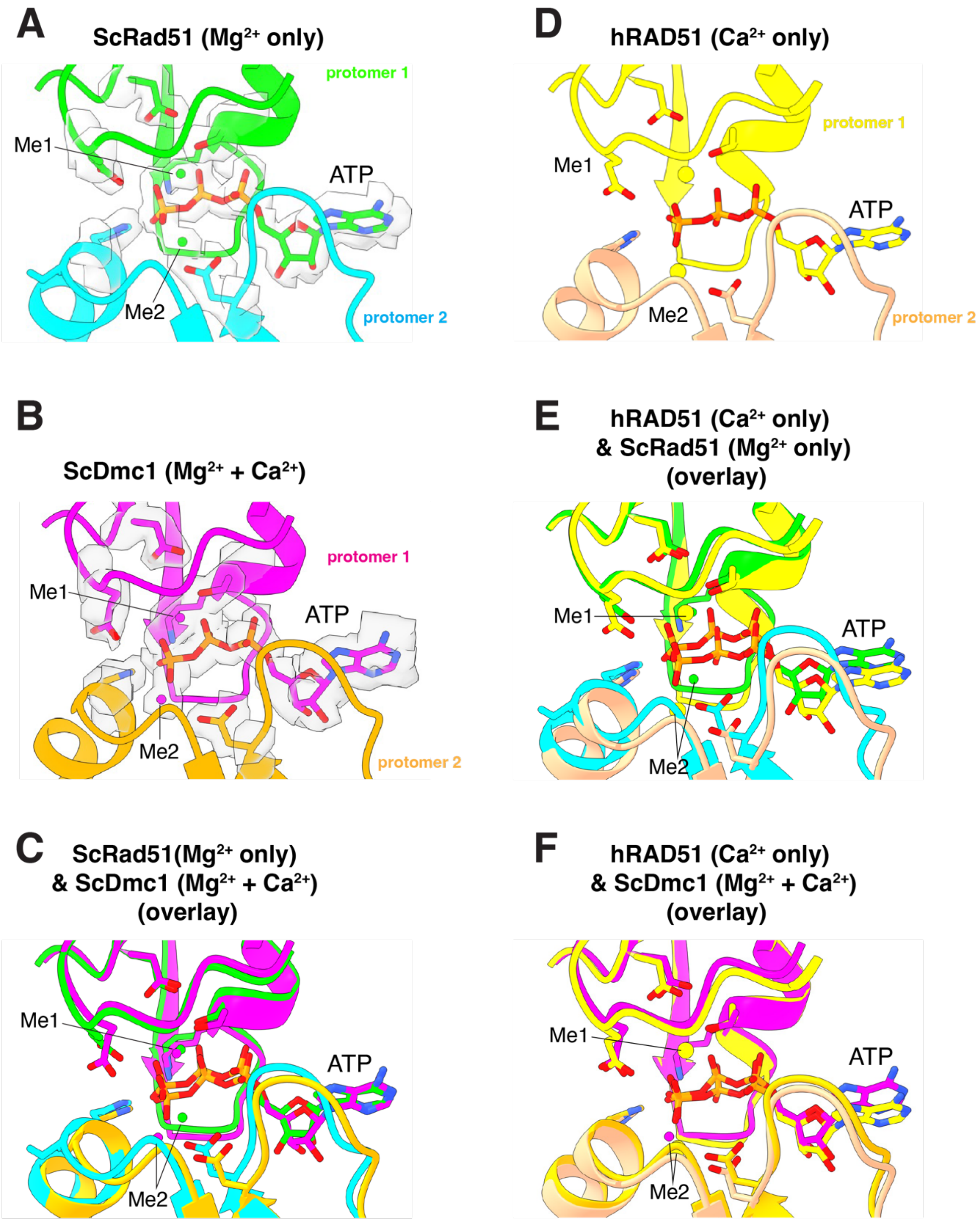

bioRxiv preprint doi: https://doi.org/10.1101/2025.03.19.644215; this version posted March 19, 2025. The copyright holder for this preprint (which was not certified by peer review) is the author/funder, who has granted bioRxiv a license to display the preprint in perpetuity. It is made available under aCC-BY 4.0 International license.

Figure S5. Differences in second metal ion positioning for samples containing Ca^{2+}. (A) ATP-binding pocket of *S. cerevisiae* Rad51 highlighting the positions of the two metal ions (PDB ID: 9D46)[44]; the sample was prepared with 20 mM Mg^{2+}. **(B)** ATP-binding pocket of *S. cerevisiae* Dmc1 highlighting the positions of the two metal ions (PDB ID: 9D4N)[44]; the sample was prepared with 20 mM Mg^{2+} plus 1.5 mM Ca^{2+}. **(C)** Overlay of *S. cerevisiae* Rad51 and Dmc1 highlighting the difference in position of the second divalent metal ion. **(D)** ATP-binding pocket of human RAD51 highlighting the positions of the two metal ions (PDB ID: 8BQ2)[45]; the sample was prepared with 5 mM Ca^{2+}. **(E)** Overlay of the ATP-binding pocket from *S. cerevisiae* Rad51 [44] and human RAD51 (PDB ID: 8BQ2)[45]. **(F)** Overlay of the ATP-binding pocket from *S. cerevisiae* Dmc1 [44] and human RAD51 (PDB ID: 8BQ2)[45]. In **(A-F)** Me1 and Me2 are used denote the first and second metal ion binding sites.

bioRxiv preprint doi: https://doi.org/10.1101/2025.03.19.644215; this version posted March 19, 2025. The copyright holder for this preprint (which was not certified by peer review) is the author/funder, who has granted bioRxiv a license to display the preprint in perpetuity. It is made available under aCC-BY 4.0 International license.

Figure S6

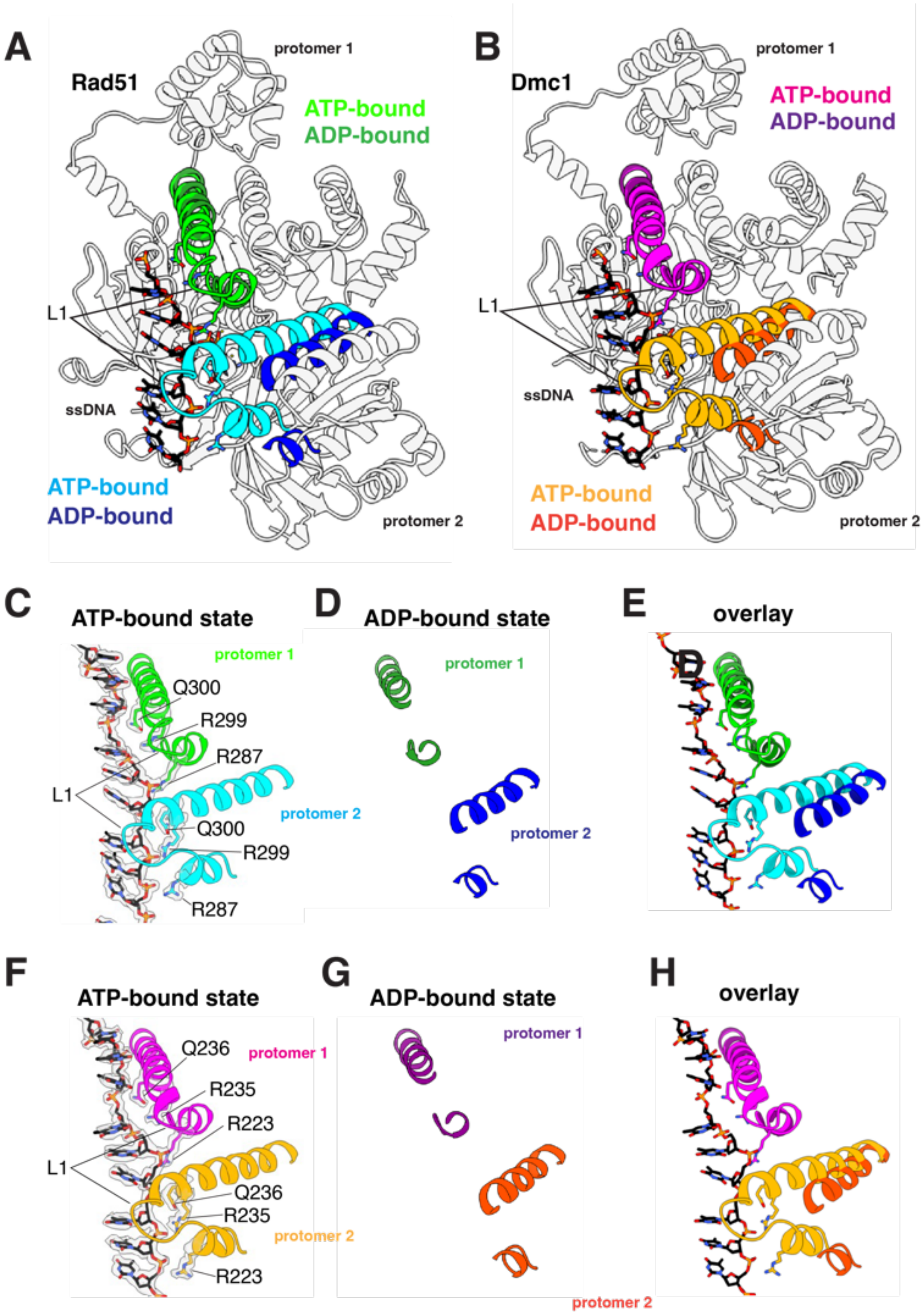

bioRxiv preprint doi: https://doi.org/10.1101/2025.03.19.644215; this version posted March 19, 2025. The copyright holder for this preprint (which was not certified by peer review) is the author/funder, who has granted bioRxiv a license to display the preprint in perpetuity. It is made available under aCC-BY 4.0 International license.

Figure S6. Loss of L1 contacts with ssDNA in the ADP-bound state. (A) Overlay of two adjacent Rad51 protomers in the ATP- and ADP-bound states, as indicated, highlighting the location of the L1 DNA-binding loop, α helix 12 and α helix 13. **(B)** Overlay of two adjacent Dmc1 protomers in the ATP- and ADP-bound states, as indicated, highlighting the location of the L1 DNA-binding loop, α helix 12 and α helix 13. **(C)** Close-up view of the Rad51 L1 contacts with the bound ssDNA substrate in the ATP-bound state. **(D)** Close-up view showing the loss of Rad51 L1 contacts with the bound ssDNA substrate in the ADP-bound state. **(E)** Overlay of the Rad51 region encompassing the L1 DNA-binding loop, α helix 12 and α helix 13 in the ATP- and ADP-bound states. **(F)** Close-up view of the Dmc1 L1 contacts with the bound ssDNA substrate in the ATP-bound state. **(G)** Close-up view showing the loss of Dmc1 L1 contacts with the bound ssDNA substrate in the ADP-bound state. **(H)** Overlay of the Dmc1 region encompassing the L1 DNA-binding loop, α helix 12 and α helix 13 in the ATP- and ADP-bound states.
